# Editorial: Metabolic Rewiring in Leukemias

**DOI:** 10.3389/fonc.2021.775167

**Published:** 2021-10-08

**Authors:** Nelida I. Noguera, Syed K. Hasan, Emanuele Ammatuna, Adriano Venditti

**Affiliations:** ^1^ Department of Biomedicine and Prevention, Tor Vergata University of Rome, Rome, Italy; ^2^ Unit of Neuro-Oncoematologia, Santa Lucia Foundation, Istituti di Ricovero e Cura a Carattere Scientifico (IRCCS), Rome, Italy; ^3^ Cell and Tumor Biology Group, Advanced Centre for Treatment, Research and Education in Cancer, Navi Mumbai, India; ^4^ Homi Bhabha National Institute, Mumbai, India; ^5^ Department of Hematology, University Medical Centre Groningen, Groningen, Netherlands

**Keywords:** metabolic plasticity, ROS, ALL and adipocytes, ketone body metabolism, dihydroorotate dehydrogenase (DHODH), tumor microenvironment, chemoresistance, leukemia

Cancer cell proliferation requires up-regulation and rewiring of metabolic pathways to feed anabolic cell growth. Oncogenic drivers directly and indirectly regulate metabolic pathways. Aberrant metabolism is central not only for leukemia cells proliferation and survival, but also mediates development of metastasis and resistance to therapies. It is now undeniable the relevant role of metabolic mechanisms in leukemogenesis with significant implications for the development of target therapies (1, 2). Depending on nutrient availability and tumor’s structural organization, some cells have a predominant glycolytic phenotype whereas others have a primarily oxidative phosphorylation (OXPHOS) metabolic phenotype. The metabolic heterogeneity defines the tumor growth organization, promoting adaptability and resistance. One of the mechanisms by which metabolic connection is established in tumors is the oxidative stress (3). It induces autophagy with mitochondrial dysfunction and shift to high rates of glycolysis (4, 5). The peculiar tumor microenvironment, characterized by hypoxia, low pH and low glucose concentrations not only shapes the metabolic phenotype of tumor cells, but interferes also with the immune response (6–8). It has been shown that leukemia cells generate significant amounts of lactate even in the presence of adequate amounts of oxygen recapitulating Warburg’s effect. The Warburg effect, could be mimicked in AML cells by mitochondrial uncoupling respiration. That forces the cells to recur to glycolysis in the absence of alterations in the oxidative capacity of cells (9). Kumar summarizes the recently published literature on the AML blasts/LSC specific activated metabolic pathways and further discusses the potential therapeutic targets for the disease cure. Leukemia cells have the ability to reduce molecular oxygen utilizing electrons from carbon sources other than pyruvate as glutamine and fatty acids (FA) (10, 11). Hematopoietic stem cells (HSCs) acquire energy mainly through anaerobic glycolysis, whereas leukemia stem cells through mitochondrial oxidative respiration (12). Mitochondria are the primary site of reactive oxygen species (ROS) generation. In this special guest edition, Robinson et al. examine the effect of cellular ROS levels on carbohydrate, lipid, and protein pathways and establish further evidence that ROS rewires metabolism in AML. They have demonstrated that ROS mediated proliferation is attributed to changes in carbohydrate metabolism, with a key glycolytic regulator, 6-phosphofructo-2- kinase/fructose-2,6-bisphosphatase 3 (PFKFB3), acting as an important mediator of ROS (13). These data provide further evidence of ROS directed metabolic changes in AML and the potential for metabolic targeting as novel therapeutic arm to combat this disease. Obesity increases the incidence and mortality of many cancers (13–15), including leukemia. In children with acute lymphoblastic leukemia (ALL), obesity increases relapse rate by ~50% (16, 17) and risk of detectible minimal residual disease after the first month of chemotherapy more than two- fold (18). Tucci et al. uncover a previously unidentified interaction between ALL cells and adipocytes, leading to transfer of free fatty acids for use as a metabolic fuel and macromolecule building block. The authors propose that this interaction may contribute to ALL resistance to chemotherapy, and could potentially be targeted to improve ALL treatment outcome. These studies add a new facet to the already complex relationship between ALL cells and adipocytes. In children, ALL risk stratification and treatment regimens have improved cure rates to nearly 90% nonetheless prognosis for relapsed remains poor. Sbirkov et al. propose a new drug, Atovaquone which is a well-tolerated drug used in the clinic mainly against malaria. This drug is a ubiquinone analogue and inhibits co-enzyme Q10 of the electron transport chain affecting OXPHOS and cell metabolism. The authors present novel data demonstrating the anti-leukemic effect of Atovaquone, the mechanism of action of the drug and the concomitant gene expression changes that may underpin the phenotypic changes observed. Importantly, an enhanced anti-leukemic effect was observed when Atovaquone was combined with the standard chemotherapeutic Idarubicin or Prednisolone. Han et al analyze key genes involved in ketone body metabolism in AML. Several studies suggest the anti- tumor effect of ketone diet (KD) in solid tumors. It is also shown that KD can improve the response of PI3K inhibitor BKM120 in MLL-AF9 AML mouse model (19). However, knowledge about the ketone metabolism in AML is very limited. Han et al. show the downregulation of key genes involved in ketone body metabolism in AML blasts as compared with normal HSCs, identify the previously unappreciated anti-tumor role of D-beta-hydroxybutyrate dehydrogenase (BDH1) in AML. This enzyme catalyzes the interconversion of acetoacetate and (R)-3-hydroxybutyrate, the two major ketone bodies produced during fatty acid catabolism. They show that low BDH1 expression predicts poor survival in AML and suggest a therapeutic potential in targeting BDH1 in the AML treatment. Recent insights into iron metabolism along with the recent discovery of ferroptosis have opened new avenues in the field of anti-tumor therapies (20, 21). Grignano et al. have reviewed the various factors involved in the physiology of iron metabolism and its deregulation in leukemia. Iron causes oxidative stress and damage, which can promote the growth and proliferation of leukemic cells. Iron metabolism is strictly regulated and the related therapeutic approaches to date have been to restrict iron availability to tumor cells. However, since a new form of iron-catalyzed cell death has been described, termed ferroptosis, iron excess is thought to represent an opportunity to selectively kill leukemic cells and spare normal hematopoietic cells, based on their differential iron needs (22). Metabolic rewiring is considered as a primary feature of cancer. Malignant cells reprogram metabolism pathway in response to various intrinsic and extrinsic drawback to fuel cell survival and growth (23, 24). Among the complex metabolic pathways, pyrimidine biosynthesis is conserved in all living organism and is necessary to maintain cellular fundamental function (i.e. DNA and RNA biosynthesis). In recent years, increased studies have evidenced the interplay of oncogenic signaling and pyrimidine synthesis in tumorigenesis. Wang et al. have elegantly reviewed the recent conceptual advances on pyrimidine metabolism, especially dihydroorotate dehydrogenase (DHODH), in the framework of precision oncology medicine and prospect how this would guide the development of new drug precisely targeting the pyrimidine metabolism in cancer.

## Author Contributions

NN and SH composed, edited, and finalized the editorial. EA and AV reviewed and finalized the editorial. All authors contributed to the article and approved the submitted version.

## Funding

This manuscript has been supported in part by Indian Council Medical Research (ICMR) grant 56/7/2019_hae-bms.

## Conflict of Interest

The authors declare that the research was conducted in the absence of any commercial or financial relationships that could be construed as a potential conflict of interest.

## Publisher’s Note

All claims expressed in this article are solely those of the authors and do not necessarily represent those of their affiliated organizations, or those of the publisher, the editors and the reviewers. Any product that may be evaluated in this article, or claim that may be made by its manufacturer, is not guaranteed or endorsed by the publisher.
